# Costs and return to scale analysis of extending the offer of pre-exposure prophylaxis (PrEP) to key populations aged 15–17 years old in two Brazilian cities

**DOI:** 10.1371/journal.pone.0332901

**Published:** 2025-10-08

**Authors:** Andreia Costa Santos, Joilson Nascimento Paim, Patricia Coelho de Soarez, Natacha Cerchiari, Laio Magno, Ines Dourado, Alexandre Grangeiro

**Affiliations:** 1 Faculty of Sports, Technology and Health Sciences, St Mary’s University, London, United Kingdom; 2 Instituto de Saúde Coletiva, Universidade Federal da Bahia, Salvador, Brazil; 3 Escola Politécnica, Universidade Federal da Bahia, Salvador, Brazil; 4 Departamento de Medicina Preventiva, Faculdade de Medicina, Universidade de São Paulo, São Paulo, Brazil; 5 Departamento de Ciências da Vida, Universidade do Estado da Bahia, Salvador, Brazil; Centers for Disease Control and Prevention, UNITED STATES OF AMERICA

## Abstract

In Brazil, HIV infection incidence is increasing, particularly among men who have sex with men (MSM) and transgender women (TGW). Pre-exposure prophylaxis (PrEP) is an effective prevention strategy, offered for free for those 15 years and over, in the Brazilian National Health System (SUS), but without a consistent demand creation strategy (DCS) to support the Sustainable Development Goals targets. The objectives of this study are to assess (1) the total incremental cost, and average total incremental costs of PrEP delivery, including DCS, targeting MSM and TGW adolescent aged 15–17 years old, and (2) the potential gains of scale for the expansion of PrEP at SUS, based on different scenarios to reach the UNAIDS goals for HIV targets. We estimated the total incremental and average total incremental cost, and the gains of scale for the expansion of PrEP delivery in SUS using Cobb-Douglas functions. The average total incremental cost per PrEP delivery was estimated at USD 321 in Salvador and USD 254 in São Paulo. Gains of scale were observed in both study settings and nationally for the Brazilian SUS. Our estimates show that investments in expanding PrEP delivery to 15–17 years old will likely reduce average total incremental costs to the Brazilian SUS. However, a cost-effectiveness analysis would be required to assess whether investments in an expansion of PrEP delivery would maximise the benefits of reducing the incidence of HIV/AIDS among the target population compared to the current Brazilian SUS practices.

## Introduction

In Brazil, an average of 40,000 new cases of HIV infections are registered annually [1,2]. Data pre-COVID-19 pandemic show steady growth in HIV infections despite the expansion of rapid HIV diagnostic testing and preventive measures, such as free distribution of condoms and lubricants, by the Brazilian National Health System (in Portuguese: *Sistema Único de Saúde –* SUS) to the population [3]. This trend has been confirmed by recent data showing an increase in HIV infections by 17% between 2020 and 2023 [4].

The epidemic of HIV in Brazil is concentrated in key populations, including men who have sex with men (MSM) and transgender women (TGW), with an estimated prevalence of 18.4% [5], and 32.1% [6], in 2016, respectively. The country’s latest available data show a worrisome statistic in AIDS incidence, particularly among 15–19 years old adolescent men, with an increased detection rate of 3.7 per 100,000 inhabitants in 2009 to 5.8 per 100,000 inhabitants in 2022, which can be explained by the increased number of infections pre-COVID-19 [1,2,4].

The expansion of HIV combination preventive strategies has been proposed to reduce infection rates by targeting 95% of key populations for access to and use of condoms and lubricants, and for those at high and very high risk of infection, also being offered pre-exposure prophylaxis (PrEP), with uptake targets of 20% for prisoners and others in closed settings and people who inject drugs, 65% for MSM and TGW, and 95% for sex workers. UNAIDS sets all these targets as part of the Sustainable Development Goal (SDG) 3, which aims to end the AIDS epidemic by 2030, for which Brazil is a signatory Member State [7].

PrEP is an effective, feasible [8], and cost-effective prevention strategy for adults over 18 years old in Brazil [9]. Since 2008, SUS has offered PrEP free of charge to those 18 years and over, but recently, it has expanded the offer to all aged 15 years and older [10]. However, the availability of free HIV combined preventive strategies, including PrEP, does not necessarily translate into high uptake of these strategies, particularly among adolescents [11,12]. Qualitative studies conducted in the country showed that the main barriers to the use of PrEP are associated with difficulties in incorporating the daily medication into individuals’ routines, its impact on their social lives (stigma) and the short-, and long-term side effects of PrEP use [13]. It is recognised that to boost demand for PrEP targeting youth and adolescents, bold strategies based on a combination of peer-facilitated interventions, social media, and face-to-face interactions are needed [13,14].

A recent study in Brazil assessed the effectiveness of demand creation strategies (DCS) for PrEP among adolescent men who have sex with men (AMSM) and adolescent transgender women (ATGW). The results showed high effectiveness in recruiting and enrolling adolescents (20.6%) into HIV preventive interventions, when a combination of DCS was implemented, although it varied by type of recruitment: direct referrals (39.5%), face-to-face peer recruitment (15.5%), and online (9.8%) [14]. Combined DCS, triage, enrolment and clinical follow-up for PrEP (e.g., HIV testing, counselling, dispensing of PrEP, and follow-up), known as PrEP delivery (a package of preventive measures), has been the strategy of focus for reducing infection rates [15].

The objective of this study is twofold: to estimate (1) the total incremental (additional) and average total incremental costs (average additional per unit) of PrEP delivery targeting AMSM and ATGW aged 15–17 years old in two large cities of Brazil, and (2) the potential gains of scale when different scenarios for the expansion of PrEP delivery to 15–17 years old target groups to reach the 65% and 95% UNAIDS goals of access, and use of HIV combination of preventive strategies for AMSM and ATGW, are assessed, in those cities.

The focus of this paper is on the costs and gains of scale for increased coverage of PrEP delivery, and it aims to provide a baseline understanding of the financial resources required and cost advantages for PrEP delivery expansion. We do not explore the health benefits of PrEP delivery. Thus, although the results presented here are useful for policymakers, they cannot inform resource allocation. A cost-effectiveness analysis based on the cost analysis of this study is being prepared for publication.

Throughout the text, we will use the expression PrEP delivery as a synonym for the expanded range of services for HIV preventive measures, including DCS.

## Materials and methods

### Intervention characteristics and settings

A demonstration study entitled PrEP15–19, aimed at assessing the effectiveness of PrEP delivery, including other combinations of HIV prevention strategies, to reduce HIV incidence among AMSM and ATGW aged between 15 and 19 years, was implemented in HIV clinic services across three capital cities in Brazil (specialised clinics) – Belo Horizonte, Salvador, and São Paulo -, from February and March 2019 to October and December 2021 [16]. Participants were recruited by trained peer educators (young MSM and TGW) and health professionals who provided information on HIV preventive methods, support to overcome stigma, and follow-up – the DCS arm of the PrEP delivery project. All strategies were informed by formative research, which included focus groups and in-depth interviews led by HIV/AIDS experts. It also provided testing for HIV, other sexually transmitted infections (STIs), and other laboratory tests, dispensing of PrEP drugs, and follow-up. Details of the program can be found elsewhere [16,17].

### Cost data collection and analysis

We collected incremental costs associated with PrEP delivery, only in the cities of Salvador and São Paulo. This economic study does not cover the city of Belo Horizonte. The incremental costs refer to the additional costs necessary to deliver PrEP, including DCS in those two cities. Salvador is located in Northeast Brazil, while São Paulo is in the Southeast region. Brazil’s north and northeast regions are economically poorer than the south and southeast. This difference is also somewhat reflected in the distribution and costs of healthcare services, including salaries paid to medical and non-medical staff, and transport costs. Our study, therefore, aimed to capture the differences in the costs of PrEP delivery in the SUS across the two settings.

Costs were estimated for 2021, and the time horizon for the analysis was five years, from 2021 to 2025. We identified the additional costs (incremental) associated with designing the DCS to be potentially implemented within SUS services. These costs include the development of the training curriculum for peer educators, additional costs with overheads for the physical adaptation of infrastructure and information systems, and the additional recruitment and training of peers and other health professionals; these costs were classified as incremental pre-implementation costs. Incremental implementation capital costs (i.e., additional furniture and equipment) and incremental recurrent costs (i.e., additional costs with overheads, medical and non-medical staff salaries, laboratory tests, drugs, and personnel working with DCS, including costs with social media, mobile phones and other associated DCS recurrent costs) were collected from the SUS database (*Banco de Preços*, in Portuguese [18]) and the Departments of Health in Salvador and São Paulo.

All costs were estimated from the perspective of SUS, by mirroring the service in practice, and by only including the additional (incremental) resources needed to expand PrEP delivery to the target population aged 15–17 years old. The DCS component is not currently part of SUS but proposed by the PrEP15–19 study. SUS does not consistently offer laboratory tests for gonorrhoea and chlamydia, although those tests are expected to be performed in the services every six months for STI management and control (S1 Fig. HIV/AIDS patients’ flow at SUS). We present the results of our analyses with and without the costs for gonorrhoea and chlamydia testing, to understand the effect of these costs on the analyses.

In Brazil, all activities associated with the payment of medical and non-medical staff, overheads, acquisition of PrEP drugs, materials, testing (e.g., HIV, other STIs, and laboratory tests), treatment (including the provision of antiretroviral therapy), and follow-up – all recurrent costs –, and capital costs are paid by SUS, at the three levels of government (i.e., municipality, states, and federal governments); no donations for any activity or products were identified, including time of experts or other personnel for the potential DCS activities. Thus, our focus on reporting the implementation recurrent costs was on the financial costs. Economic (opportunity) costs, with experts’ incremental time for the formative research and development of the training programme, were captured in the pre-implementation costs. Pre-implementation costs only include the costs of developing the training materials (including formative research as an integral part of the development of the DCS) and personnel training in DCS.

Pre-implementation and capital implementation incremental costs were annualised using a discount rate of 3% and a five-year life expectancy, to account for the depreciation of goods and services [19]. We did not discount other costs to account for time preferences [19]. All costs were adjusted by inflation (through 2025) using an average of the expanded consumer price index (IPCA in Portuguese) as calculated by the Brazilian government for 2020–2023 and estimated at 3.5%, annually [20]. Costs were estimated in 2021 Brazilian Real (R$) and US Dollars (USD), using an average exchange rate of 5.156 for 2021 (Historical Currency Converter | OANDA). Total incremental costs and average total incremental costs of PrEP delivery were estimated for the cities of Salvador and São Paulo, then extrapolated to Brazil, as an average cost for the two cities, for the scaling-up analysis.

### Gains of scale for the expansion of PrEP delivery to SUS

We used a Cobb-Douglas production function [21] (see S2 File for more details) to assess gains of scale in expanding PrEP delivery to the target populations in Salvador, São Paulo, and Brazil. In economics, gains of scale (or economies of scale) represent a situation where long-run average costs decrease as the quantity of the output (coverage) increases. On the other hand, diseconomies of scale occur when long-run average costs increase as output increases, while constant economies of scale represent the situation where the long-term average costs do not change as output increases [21]. For this analysis, we used population data for 2021 as a baseline, for the age group 15–17 years old, and population growth rates, obtained from the Brazilian Institute of Geography and Statistics (IBGE), to assess population coverage [22]. Estimates on the proportion of MSM and TGW in the population were obtained from the IBGE’s National Health Statistics (PNS) for 2019 [23] and from the literature [24], and extrapolated to the target age groups (AMSM and ATGW) since we do not have available statistics for this specific age group.

We assessed two exclusive main scenarios (not occurring simultaneously) for the scaling-up analysis, and both are anchored on the assumption that DCS would yield an increase in demand for PrEP, helping achieve UNAIDS targets:

**Scenario one**: Departing from the estimated population of AMSM and ATWG in 2021 – baseline -, and costs estimates for the PrEP delivery, we assessed gains of scale in expanding PrEP delivery for coverages of 40%, 50%, 65% (UNAIDS global target), 85% and 95% (UNAIDS risk groups target) of this population group from 2021–2025. As coverage increases, we also assumed an annual 5% reduction in incremental implementation recurrent costs, yielding an approximately 28% reduction in this cost category in 5 years. This is a conservative assumption based on our assessment and evidence from the literature [25] that PrEP delivery is underused in Brazil. It can reach a higher demand than what they are currently covering, thus maximising efficiency for SUS. It is expected that an increase in demand will allow for a more efficient use of human and other financial resources, potentially reducing the average total incremental recurrent costs of DCS and other activities, although the total incremental recurrent cost would increase because of the increased coverage. Of course, cost variations are expected depending on the combinations of DCS chosen for implementation; however, previous studies (in other contexts) suggest evident economies of scale with unit costs lower in sites when higher numbers of clients are reached [26].

**Scenario two**: In this scenario, we assumed a reduction of 7% in the costs of PrEP drugs while the remaining implementation recurrent costs are reduced at 5%, as in scenario 1. In Brazil, oral PrEP drugs, combining tenofovir (TDF) and emtricitabine (FTC), have been acquired in bulk from the pharmaceutical industry with the commercial name Truvada®. The price of Truvada®, per pill, has decreased from R$ 2.63 to R$ 2.25 (USD 0.51 to USD 0.44, in 2021 prices), from 2017 to 2020, a 14.45% reduction in price for the period [27]. Further reductions in the price of oral PrEP drugs to SUS are expected, as national production by a Brazilian public Laboratory (Farmanguinhos) started in 2020 [28]. In our analysis, the 7% decrease in prices of PrEP drugs (half of the observed real reduction in prices between 2017 and 2020 for the Truvada®) is a conservative assumption used to assess the impact of expanding PrEP delivery to achieve UNAIDS targets [29].Gains of scale were assessed for the two scenarios described above and presented as an analysis for when all cost estimates were included and for when costs of testing for gonorrhoea and chlamydia were excluded, as SUS does not consistently offer these tests in its services.

### Sensitivity analysis

All cost data were obtained from SUS databases, and the project accounts specifically for the incremental *time* of professionals to develop the DCS programme, with the costs for these professionals based on SUS prices. The main sources of uncertainty for the costs are related to the discount rates used, as they directly affect the cost estimation for the main components of demand creation (incremental pre-implementation costs and incremental implementation capital costs). Using tornado diagrams for one-way sensitivity analysis, we varied the discount rate used for those cost categories in 0% and 10% (baseline = 3%), to assess the role of these parameters in the total incremental and average total incremental cost. We also tested scenario 1 for a reduction of 2.5% in implementation incremental costs instead of 5%, and scenario 2 for reductions of 3.5% in PrEP drugs, instead of 7%, and 2.5% in all other implementation incremental costs, instead of 5%.

### Ethics

This study was conducted following the principles of the Brazilian Research Ethics Commission (CONEP), which complies with all Brazilian laws on adolescents’ rights, and the Declaration of Helsinki. The research protocol was approved by the World Health Organization’s Ethics Review Committee (protocol ID: Fiotec-PrEP Adolescent study), and by the Ethics Review Committee from the study coordinating universities: University of São Paulo (#3,082,360), in December 2018, and the Federal University of Bahia (#3,224,384), in March 2019. Field work for the project took place from February 2019 to October 2021 in São Paulo and from April 2019 to December 2021 in Salvador. The city of Belo Horizonte was not part of this costing study. Participation was voluntary, and all collected data were kept confidential. An informed consent form explaining the research aims and procedures, and participants’ rights, was presented to each participant before they decided to participate in this study. Special judicial authorization was obtained to allow for waiver of parental consent for individuals aged under 18 in São Paulo and Salvador. The waiver applied to adolescents deemed at risk of violence related to sexual and gender identity disclosure and in cases of the breakage of family ties.

Additional information regarding the ethical, cultural, and scientific considerations specific to inclusivity in global research is included in the Supporting Information (S3 File PLOS’ questionnaire on inclusivity).

## Results and discussion

The total incremental cost of PrEP delivery, including DCS, for Salvador was estimated at USD 60,370 (R$ 310,387), and USD 79,265 (R$ 408,689) for São Paulo, in 2021, with an average total incremental cost per patient of USD 321 and USD 254, in the respective settings (Table 1). Considering that SUS does not regularly offer gonorrhoea and chlamydia testing, we estimated the average total incremental cost of PrEP delivered per patient at USD 297, in Salvador, and USD 238 in São Paulo, when those costs are excluded (Table 1). The average total incremental cost of PrEP delivery for SUS was estimated at USD 287 and USD 268 in Brazil (a simple average for the costs in Salvador and São Paulo), for when all costs were included and when costs of gonorrhoea and chlamydia testing were excluded, respectively.

**Table 1:** Total incremental and average incremental total costs of PrEP delivery to adolescents aged 15-19 years old in Salvador and São Paulo, 2021 prices.

	Salvador			São Paulo		
**Incremental pre-implementation costs – set up phase**	R$	US$	%	R$	US$	%
**TOTAL pre-implementation incremental costs**	19,724	3,826	6%	26,689	5,176	7%
**Developing training material**	12,568	2,438	64%	17,532	3,400	66%
**Experts time**	6,812	1,321	54%	8,175	1,585	47%
**Consultancies**	2,688	521	21%	3,011	584	17%
**Equipment and materials**	1,482	287	12%	1,749	339	10%
**Overheads**	1,585	307	13%	4,598	892	26%
**Formative research**	4,872	945	25%	6,187	1,200	23%
**Demand creation (training)**	2,285	443	12%	2,970	576	11%
**Incremental implementation costs**	R$	US$	%	R$	US$	%
**TOTAL incremental implementation capital costs**	21,598	4,189	7%	42,056	8,157	10%
**Furniture**	4,110	797	19%	8,632	1,674	21%
**Equipment (infrastructure: e.g., air conditioning)**	3,138	609	15%	5,648	1,095	13%
**Equipment work (e.g., computer, printer, etc.)**	13,498	2,618	62%	25,646	4,974	61%
**Equipment (laboratory)**	852	165	4%	2,130	413	5%
**TOTAL incremental implementation recurrent costs**	269,065	52,356	87%	339,944	66,030	83%
**Overheads**	1,026	199	0.4%	1,538	298	0.5%
**Health professionals (including lab staff)**	17,850	3,462	7%	19,854	3,851	6%
**Administrative Staff**	2,464	478	1%	2,772	538	1%
**Syphilis, hepatitis B & C, renal & hepatic functions tests**	35,348	6,856	13%	45,048	8,737	13%
**Gonorrhoea & chlamydia tests**	23,460	4,550	9%	24,420	4,736	7%
**PrEP drugs**	13,950	2,706	5%	89,528	17,364	26%
**Other (condoms, HIV rapid tests, lubricating jelly, etc.)**	33,125	6,596	13%	10,633	2,160	3%
**Transport**	15,175	2,943	6%	6,321	1,226	2%
**Demand creation – personnel**	116,400	22,576	43%	129,630	25,142	38%
**Demand creation – social media, mobile phones, internet**	10,268	1,991	4%	10,200	1,978	3%
**TOTAL INCREMENTAL COST (including all cost categories)**	310,387	60,370	100%	408,689	79,265	100%
**Number of patients**	188	188	–	313	313	–
**Average incremental total cost per patient (including all cost categories)**	1,651	321	–	1,306	254	–
**TOTAL INCREMENTAL IMPLEMENTATION RECURRENT COSTS (excluding costs with gonorrhoea & chlamydia tests)**	286,927	55,821	–	384,269	74,626	–
**Number of patients**	188	188	–	313	313	–
**Average total incremental implementation recurrent cost per patient (excluding costs with gonorrhoea & chlamydia tests)**	1,526	297	–	1,228	238	–

Incremental pre-implementation costs were estimated at USD 3,826 and USD 5,176, representing 6% and 7% of the total costs, with experts’ time and consultancies corresponding to 54% and 47% of the developing training materials category, in the respective cities (Table 1). For the implementation phase of the PrEP delivery, incremental implementation recurrent costs responded for the bulk of total costs: in Salvador, 87%, and São Paulo, 83% of the total costs, with personnel’s cost to work on DCS representing 43% of the total incremental implementation recurrent costs, in Salvador, and 38% in São Paulo. Testing for syphilis, hepatitis B & C, and renal & hepatic functions corresponded to 13% of the total incremental implementation recurrent costs in the two cities, followed by the distribution of HIV self-test, HIV rapid tests, condoms, and other products, with Salvador having a higher proportion of these costs (13%) compared to São Paulo (3%). On the other hand, São Paulo had higher costs with PrEP drug distribution (26%) compared to Salvador (5%). Incremental implementation capital costs corresponded to 7% and 10% of the total costs in Salvador and São Paulo, respectively, with most of these costs spent on equipment work, such as computers, printers, etc. (Table 1).

Increasing the coverage of PrEP delivery to reach the UNAIDS targets of 65% and 95% in five years, and assuming reductions of 5% in incremental implementation recurrent costs (scenario 1), would generate gains of scale in the three settings: 1.110 (95% CI: 1.058; 1.163), for Salvador, 1.106 (95% CI: 1.028; 1.184), for São Paulo, and 1.111 (95% CI: 1.085; 1.137), for Brazil, when all costs categories are accounted for (Table 2). Economies of scale were also observed when costs for gonorrhoea and chlamydia tests were excluded from the incremental implementation recurrent costs: 1.157 (95% CI: 1.117; 1.198), 1.166 (95% CI: 1.123; 1.215), and 1.163 (95% CI: 1.121; 1.217), for São Paulo, Salvador, and Brazil, respectively (Scenario one, Table 2).

**Table 2:** Estimated gains of scale coefficients* for 95% confidence interval (CI) based on three scenarios for Salvador, São Paulo and Brazil, 2021-2025.

	Scenario 1 – increase coverage and reduce incremental implementation recurrent costs by 5%	
	Average total incremental costs (all costs)	Average total incremental costs (excluding gonorrhoea and chlamydia costs)
**Settings**	***β***_***1***_*** + β***_2_^**^ ***(95% CI)***^^^	***β***_***1***_*** + β***_2_^**^ ***(95% CI)***^^^
Salvador	1.110 (1.058; 1.163)	1.157 (1.117; 1.198)
São Paulo	1.106 (1.028; 1.184)	1.166 (1.123; 1.215)
Brazil	1.111 (1.085; 1.137)	1.163 (1.121; 1.217)
	Scenario 2 – increase coverage and reduce PrEP drug costs by 7% and incremental implementation recurrent costs by 5%	
	Average total incremental costs (all costs)	Average total incremental costs (excluding gonorrhoea and chlamydia costs)
**Settings**	***β***_***1***_*** + β***_2_^**^ ***(95% CI)***^^^	***β***_***1***_*** + β***_2_^**^ ***(95% CI)***^^^
Salvador	1.147 (1.067; 1.228)	1.209 (1.144; 1.275)
São Paulo	1.145 (1.024; 1.266)	1.216 (1.154; 1.278)
Brazil	1.152 (1.111; 1.194)	1.212 (1.149; 1.276)

*Coefficients <1 indicates diseconomies of scale, coefficients = 1 indicates constant gains of scale, while coefficients > 1 indicates gains of scale

**Elasticity coefficients

^*p*-value < 0.001

Further gains of scale would be observed when a 7% reduction in the costs of PrEP drugs and a 5% reduction of the remaining incremental implementation recurrent costs were assumed, with gains of scale estimated at 1.147 (95% CI: 1.067; 1.228), for Salvador, 1.145 (95% CI: 1.024; 1.266), for São Paulo, and 1.152 (95% CI: 1.111; 1.194) for Brazil, when assessing all costs, while for when costs for gonorrhoea and chlamydia tests were excluded, gains of scale of 1.209 (95% CI: 1.144; 1.275), 1.216 (95% CI: 1.154; 1.278) and 1.212 (95% CI: 1.149; 1.276), respectively for Salvador, São Paulo, and Brazil would be observed (Scenario two, Table 2).

**Fig 1:**
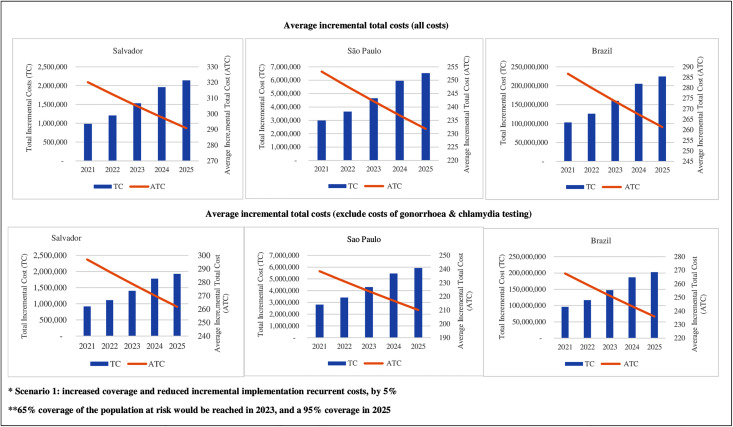
Total incremental and average incremental total costs of PrEP delivered scaling-up scenario 1 for Salvador, São Paulo and Brazil (2021-2025).

**Fig 2:**
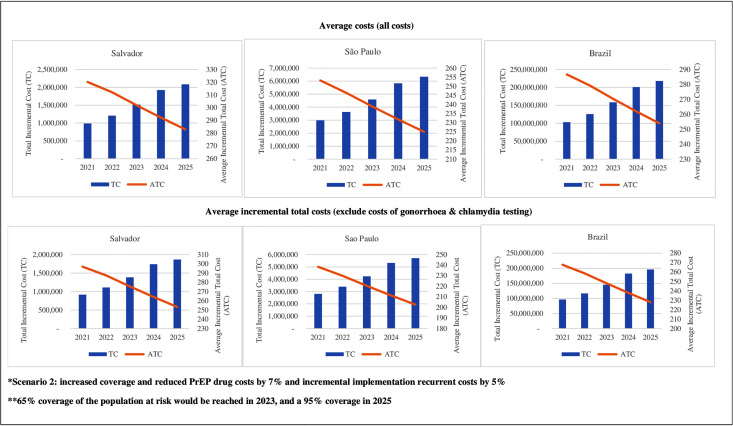
Total incremental and average incremental total costs of PrEP delivered at scaling-up scenario 2 for Salvador, São Paulo and Brazil (2021-2025).

If economies of scale are observed in the three settings, average total incremental costs are expected to decrease while coverage and total incremental costs increase (Figs 1 and 2). For Salvador, to reach a coverage of PrEP delivery of 65% of the target population, in 2023, and for a decrease in incremental implementation recurrent cost by 5% (scenario 1), the average total incremental cost was estimated at USD 302, at a total incremental cost of USD 1.5 million. In São Paulo, for the same scenario 1, average total incremental costs for a 65% coverage of the target population were estimated at USD 238, with total incremental costs of USD 4.6 million, while for Brazil, these costs were USD 270 and USD 158 million, respectively, when all costs were included (Fig 1 and S4 Table). For a coverage of 95% in 2025, and considering scenario 1, the average total incremental cost would be of USD 283, USD 224 and USD 253, and total incremental costs of USD 2.1 million, USD 6.3 million and USD 217.5 million, when all costs were assessed, in Salvador, Sao Paulo and Brazil, respectively (Fig 1 and S4 Table – scenario 1).

When deducting the costs of gonorrhoea and chlamydia tests from the total costs, average total incremental costs and total incremental costs were estimated at USD 279, USD 224 and USD 251, and US$ 1.4 million, USD 4.3 million and USD 147.2 million, for a coverage of 65%, while that for a coverage of 95%, costs average total incremental costs were estimated at USD 262, USD 210 and USD 236, and USD 1.9 million, USD 5.9 million and USD 202.6 million, respectively, to Salvador, São Paulo and Brazil, for scenario 1 (Fig 1 and S4 Table – scenario 1).

When PrEP drugs were reduced by 7% and incremental implementation recurrent costs by 5% (scenario 2), total incremental and average incremental total costs in Salvador, São Paulo, and Brazil for a coverage of 65% would be USD 1.3 million, USD 3.7 million and USD 132.8 million, and USD 258, USD 195, USD 227, respectively, and USD 1.8 million, USD 5 million and USD 178.9 million, and USD 238, USD 179, and USD 208, respectively, for the a coverage of 95%, and when all costs are considered (Fig 2 and S4 Table – scenario 2). Excluding gonorrhoea and chlamydia testing costs, total incremental costs for a 65% coverage would range from USD 1.4 million, USD 4.2 million, and USD 145.3 million, an average incremental total cost from USD 276, USD 220 and USD 248, respectively, in Salvador, Sao Paulo and Brazil, in scenario 2. Total incremental costs for scenario 2 and a coverage of 95% would vary from USD 1.9 million, USD 5.7 million and USD 195.8 million, and average incremental total costs from USD 253, USD 203 and USD 228, in Salvador, Sao Paulo and Brazil, respectively (Fig 2 and S4 Table – scenario 2).

**Fig 3:**
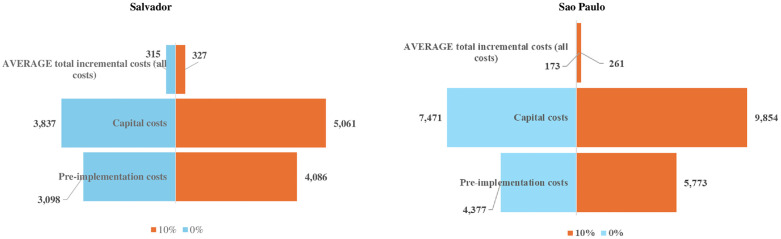
Sensitivity analysis: varying the discount rates to 0% and 10% in Salvador and São Paulo, 2021.

Average total costs varied from USD 315 to USD 327 (reference average total cost of USD 321), in Salvador, and USD 173 and USD 261 (reference average total cost of USD 254), in São Paulo, for zero and ten percent discount rates, respectively, in the sensitivity analysis, for when all costs were considered (Fig 3). Gains of scale were observed even when the assumption for the proportions of cost reductions in scenarios 1 and 2 was halved, from 5% to 2.5%, in scenario 1, and from 7% and 5% to 3.5% and 2.5%, in scenario 2, respectively, although large 95% confidence intervals were estimated for the average total costs, when all costs were inputted (S5 Table).

## Conclusions

This costing study aimed to provide decision-makers with information about the total incremental and average total incremental costs of increasing coverage of PrEP delivery and its potential gains from scale. Gains of scale functions are useful studies to assess the relationship between output (coverage) and production inputs (PrEP delivery). It does not examine the relationship between the number of infections averted or the number of people enrolled (outcome or output) and PrEP delivery (inputs), which is only possible through a cost-effectiveness analysis. A combination of assessments of gains of scale and cost-effectiveness analysis is a cornerstone of economic analysis [30].

The average total incremental annual cost per PrEP delivered, including DCS, with or without gonorrhoea and chlamydia testing are expected to decrease over time, bringing gains of scale to SUS. The reasons for this are based on evidence that PrEP services are underused [25], and PrEP drugs are expected to be reduced in price, as national production starts [28]. Evidence from the literature on better procurement management and better use of services also supports these arguments [12,29,31]. However, these gains in scale would only be realised if DCS can identify, retain, and follow up individuals through their pathway in SUS [14,25]. The current effectiveness rates of DCS in the Brazilian context need to be assessed in a cost-effectiveness analysis for its value for money and to better inform resource allocation to policymakers.

Differences in incremental implementation recurrent costs were evident in the distribution of condoms, HIV self-test and other HIV rapid tests, lubricating jelly, and other protective measures – which in Salvador was more intense -, and the delivery of PrEP drugs – which São Paulo had a higher prescription. Although SUS provides a clear flow for patients attending HIV/AIDS specialised clinics, including defining the type and number of tests needed for each patient visit (see S1 Fig. HIV/AIDS patients’ flow at SUS), providers would vary in their delivery of PrEP, depending on the local prevalence of STIs, although no differences in testing costs were observed in the two settings.

Gains of scale suggested by our analysis could be further observed with the expansion of PrEP delivery to primary health care and community services, furthering the prescription of PrEP drugs beyond medical doctors to include nurses and other health professionals [15,16]. The expansion of PrEP delivery would not only save resources for SUS with ART drugs, human resources, and overheads, when expanding this intervention, but also directly impact the incidence rates of HIV.

As previously stated, gains of scale due to increases in coverage and reduction of costs would be possible only if the demand for PrEP delivery increases. Studies in Brazil showed that barriers to PrEP acceptance include stigma and difficulties in including PrEP into individuals’ routines [13,14]. The consistent implementation of DCS encompassing a range of strategies is fundamental for the success of PrEP delivery. Investments in the expansion of bold DCSs targeting key populations and age groups should be a priority for the success of the UN SDG 3 to end the HIV endemic by 2030 [32].

Studies on the assessment of gains of scale for PrEP are uncommon in the literature, with studies of this type mostly published for the scaling-up of HIV testing. d’Elbée and colleagues assessed the costs and gains of scale for HIV testing for key populations in Côte d’Ivoire, Senegal, and Mali, and estimated a return to scale for the expansion of HIV testing in the three settings (no DCSs included) [33]. The scenario considered in their study included higher annual cost reductions for the distribution of tests and headquarters costs (compared to our analysis), with reductions varying from 20% to 85%, from 2021 to 2023 (baseline in 2020) [33]. Differently from Brazil, these countries relied heavily on donations and international consultancy costs, which can be drastically reduced when scaled at the national level.

Our cost estimates are comparable to other studies conducted in similar income level contexts as in Brazil, an upper-middle income country. In Thailand, costs per PrEP delivered were estimated, for two hospitals, at USD 223 and USD 311, in 2016 prices [34]. But it was unclear what type of laboratory or rapid tests (HIV, other STDs, and other blood tests) were offered to patients, for the analysis in Thailand. Likely, the structure, organisation, and delivery of HIV/AIDS services differ in the two countries; thus, differences in the use of resources for the delivery of PrEP should be observed. In South Africa, another comparable income context, the cost per PrEP delivered was estimated at USD 150 (2007 prices), where the bulk of costs corresponded to the PrEP drugs (89%) and the remaining costs to HIV counselling and testing, and serum creatinine testing [35].

Some limitations should be considered in our cost estimates. Although we have assessed the incremental costs of PrEP delivery expansion in two Brazilian cities with different socio-demographic characteristics, services in Brazil vary greatly in terms of quality and access. Thus, our estimates should be taken with caution, for cost extrapolations. Special attention should be given to the process of DCS, including the costs of telecommunication across regions of the country.

Despite the limitations, our estimates show that investments in expanding PrEP delivery to the Brazilian SUS for AMSM and ATGW aged 15–19 years old to reach the 65% and 95% UNAIDS targets are likely to reduce average total costs over time, with gains in scale even when more conservative reduction in costs were assumed (see sensitivity analysis). However, a cost-effectiveness analysis would be required to assess whether investments in an expansion of PrEP delivery would maximise the benefits of reducing the incidence of HIV/AIDS among the target population compared to the current Brazilian SUS practices. Our estimates provide a guide for the costs of further investments in the prevention and management of HIV/AIDS.

## Supporting information

S1 Fig Patient Flow HIV/AIDS at SUS. (TIFF)

S2 File Cobb-Douglas production function. (DOCX)

S3 File PLOS’ questionnaire on inclusivity. (DOCX)

S4 Table Total incremental and average total incremental costs. (DOCX)

S5 Table Sensitivity analysis: estimated gains of scale coefficients based on three scenarios for Salvador, São Paulo and Brazil, 2021–2025. (DOCX)

S6 File Additional information: costs used in the analysis. (XLSX)
