# Supplementary figures and images for: Costs and return to scale analysis of extending the offer of pre-exposure prophylaxis (PrEP) to key populations aged 15–17 years old in two Brazilian cities
Source: PLoS One. 2025 Oct 8;20(10):e0332901. doi: 10.1371/journal.pone.0332901 (PMC12507317; doi:10.1371/journal.pone.0332901)

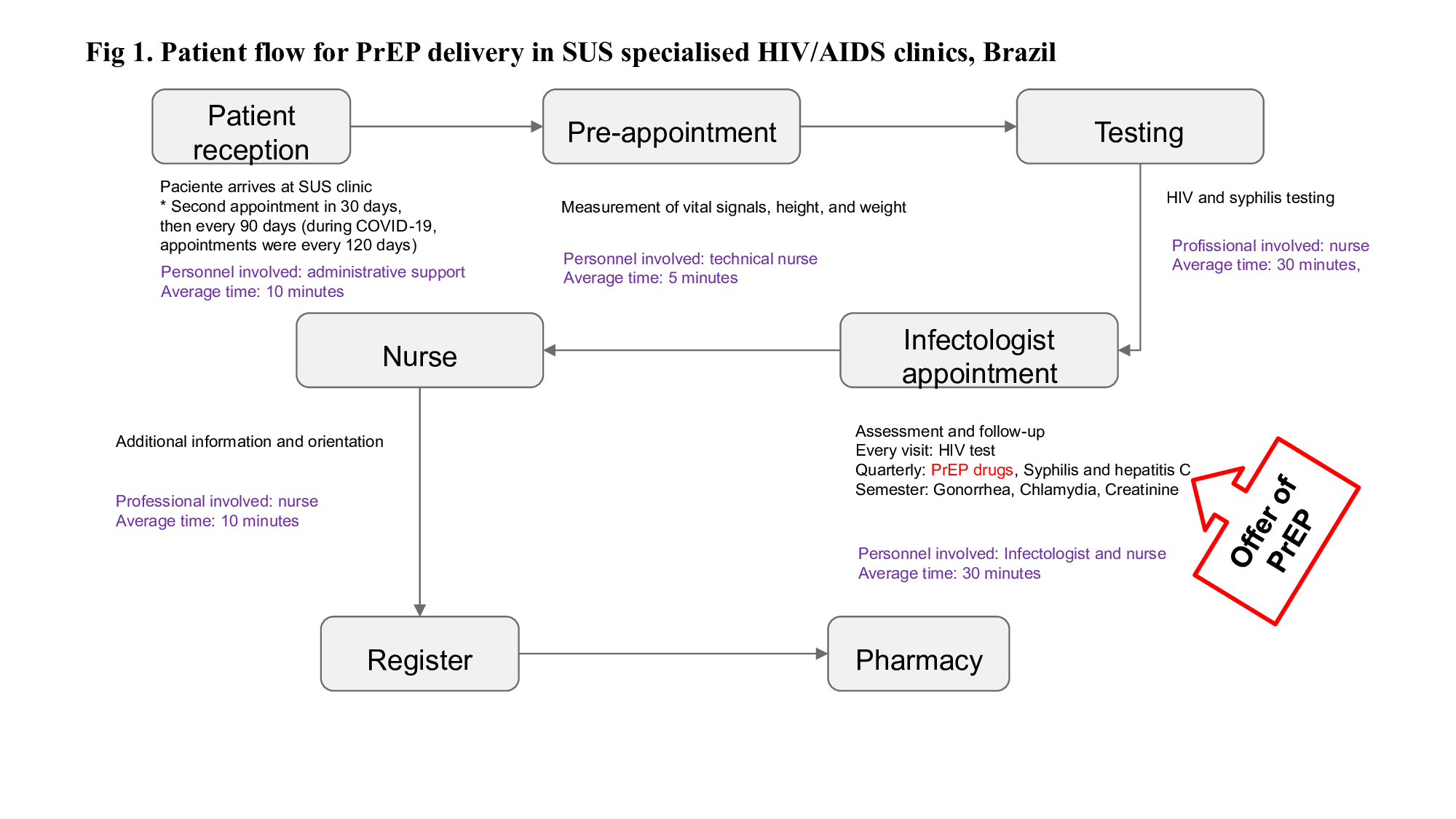

Supplement: S1 Fig — (TIFF) [file pone.0332901.s001.tiff]
