# Supplementary material for: Costs and return to scale analysis of extending the offer of pre-exposure prophylaxis (PrEP) to key populations aged 15–17 years old in two Brazilian cities
Source: PLoS One. 2025 Oct 8;20(10):e0332901. doi: 10.1371/journal.pone.0332901 (PMC12507317; doi:10.1371/journal.pone.0332901)
Supplement: S2 File — (DOCX) [file pone.0332901.s002.docx]

**Cobb-Douglas production function**

The Cobb-Douglas function is presented below (Equation 1), as well as its related functions [1]. The Cobb-Douglas function describes the relationship between outputs (quantity, *Q*) and inputs (labour (*L*) and capital (*K*)), with *β* representing the responsiveness of the output to a change in levels of inputs, i.e., elasticity of productivity, where *β_1_* is the elasticity of labour and *β_2_* is the elasticity for capital. If *β_1_+β_2_* = 1, the production displays constant return to scale, meaning that doubling inputs the same would be observed for outputs; if *β_1_+β_2_* > 1 it shows increased return to scale, which means that a percentage increase in inputs will produce a larger percentage increase in outputs, while, *β_1_+β_2_* 1 shows a decrease return to scale, meaning that a percentage increase in inputs will produce a smaller percentage increase in outputs [1,2]. In assessing scaling-up of PrEP interventions scenarios with *β_1_+β_2_* > 1, would indicate the most efficient production levels for gains of investments in PrEP.

$Q_{j}=L_{j}^{\beta_{1}}K_{j}^{\beta_{2}}$ (1)

Where, ***j*** = level of analysis (Salvador, São Paulo, Brazil).

As in the short run, capital costs are fixed ($\bar{K}$), thus:

$L_{j}=\frac{Q_{j}^{1/{\beta_{1}}}}{\bar{K}_{j}^{{\beta_{2}}/{\beta_{1}}}}$ (1.1)

The total incremental cost (*TC*) is defined as the sum of variable incremental costs (*VC*) and fixed incremental costs (*FC*) [3], we have:

${TC}_{j}= {VC}_{j}+ {FC}_{j}$ (2)

with

${VC}_{j}={UC}_{j}.S_{j}$ (2.1)

Where:

***UC_j_* =** Unit cost per variable inputs *j* for one output (e.g., HIV tests, PrEP drugs, etc.).

***S_j_*** = Scale variable for input j to reach desired number of outputs.

Relating equations (1) and (2), we have:

${TC}_{j}=\frac{\left( {UC}_{j}xS_{j} \right).\left( Q_{j}^{1/{\beta_{1}}} \right)}{\bar{K}_{j}^{{\beta_{2}}/{\beta_{1}}}}.\bar{K}$ (3)

With ${ATC}_{j}=\frac{{TC}_{j}}{Q_{j}}$ (3.1), where ***ATC*** is the average incremental total cost.

**References**

1. Cobb C, Douglas P. Theory of Production. Amer Econ Review. 1928;18: 139–65.

2. Jondrow J, Knox Lovell CA, Materov IS, Schmidt P. On the estimation of technical inefficiency in the stochastic frontier production function model. Journal of Econometrics. 1982;19: 233–238. doi:10.1016/0304-4076(82)90004-5

3. d’Elbée M, Traore MM, Badiane K, Vautier A, Simo Fotso A, Kabemba OK, et al. Costs and Scale-Up Costs of Integrating HIV Self-Testing Into Civil Society Organisation-Led Programmes for Key Populations in Côte d’Ivoire, Senegal, and Mali. Front Public Health. 2021;9: 653612. doi:10.3389/fpubh.2021.653612
