# Supplementary material for: Costs and return to scale analysis of extending the offer of pre-exposure prophylaxis (PrEP) to key populations aged 15–17 years old in two Brazilian cities
Source: PLoS One. 2025 Oct 8;20(10):e0332901. doi: 10.1371/journal.pone.0332901 (PMC12507317; doi:10.1371/journal.pone.0332901)
Supplement: S4 Table — (DOCX) [file pone.0332901.s004.docx]

**S4 Table. Total incremental and average total incremental costs**

| **Scenario 1 - increase coverage and reduce incremental implementation recurrent costs by 5%** | | | | | | | |
| --- | --- | --- | --- | --- | --- | --- | --- |
|  |  | **Salvador** | | **Sao Paulo** | | **Brazil** | |
| **Year** | **Coverage (%) target population** | **Total incremental cost** | **Average total incremental cost** | **Total incremental cost** | **Average total incremental cost** | **Total incremental cost** | **Average total incremental cost** |
| 2021 | 40 | 989,763 | 321 | 2,988,921 | 254 | 103,266,914 | 287 |
| 2022 | 65 | 1,200,648 | 311 | 3,625,761 | 246 | 125,269,673 | 278 |
| 2023 | 75 | 1,514,725 | 302 | 4,574,222 | 238 | 158,038,910 | 270 |
| 2024 | 85 | 1,922,269 | 292 | 5,804,938 | 231 | 200,560,002 | 261 |
| 2025 | 95 | 2,084,940 | 283 | 6,296,178 | 224 | 217,532,292 | 253 |
| **Scenario 2 - increase coverage and reduce PrEP drug costs by 7% and incremental implementation recurrent costs by 5%** | | | | | | | |
|  |  | **Salvador** | | **Sao Paulo** | | **Brazil** | |
| **Year** | **Coverage (%) target population** | **Total incremental cost** | **Average total incremental cost** | **Total incremental cost** | **Average total incremental cost** | **Total incremental cost** | **Average total incremental cost** |
| 2021 | 40 | 989,763 | 321 | 2,988,921 | 254 | 103,266,914 | 287 |
| 2022 | 65 | 1,040,125 | 270 | 2,999,929 | 203 | 106,370,872 | 236 |
| 2023 | 75 | 1,298,398 | 258 | 3,744,840 | 195 | 132,783,787 | 227 |
| 2024 | 85 | 1,630,393 | 248 | 4,702,381 | 187 | 166,736,085 | 217 |
| 2025 | 95 | 1,749,750 | 238 | 5,046,630 | 179 | 178,942,400 | 208 |
