# Supplementary material for: Costs and return to scale analysis of extending the offer of pre-exposure prophylaxis (PrEP) to key populations aged 15–17 years old in two Brazilian cities
Source: PLoS One. 2025 Oct 8;20(10):e0332901. doi: 10.1371/journal.pone.0332901 (PMC12507317; doi:10.1371/journal.pone.0332901)
Supplement: S5 Table — (DOCX) [file pone.0332901.s005.docx]

**S5. Sensitivity analysis: estimated gains of scale coefficients* based on three scenarios for Salvador, São Paulo and Brazil, 2021-2025**

|  | **Scenario 1 - increase coverage and reduce incremental implementation recurrent costs by 5%** | | |
| --- | --- | --- | --- |
|  | **Average incremental costs (all costs)** |  | **Average incremental costs (excluding gonorrhoea and chlamydia costs)** |
| **Settings** | ***β_1_+β_2_*** *(95% CI)*** |  | ***β_1_+β_2_*** *(95% CI)*** |
|  |  |  |  |
| Salvador | 1.02 (-1.763; 3.803) |  | 1.024 (1.019; 1.030) |
| São Paulo | 1.019 (-1.131; 3.170) |  | 1.025 (1.018; 1.031) |
| Brazil | 1.019 (-1.449; 3.489) |  | 1.024 (1.018; 1.031) |
|  | **Scenario 2 - increase coverage and reduce PrEP drug costs by 7% and incremental implementation recurrent costs by 5%** | | |
|  |  |  |  |
|  | **Average incremental costs (all costs)** |  | **Average incremental costs (excluding gonorrhoea and chlamydia costs)** |
| **Settings** | ***β_1_+β_2_*** *(95% CI)*** |  | ***β_1_+β_2_*** *(95% CI)*** |
|  |  |  |  |
| Salvador | 1.021 (-0.043; 2.084) |  | 1.044 (1.029; 1.058) |
| São Paulo | 1.026 (0.202; 2.254) |  | 1.046 (1.033; 1.058) |
| Brazil | 1.023 (0.061; 1.985) |  | 1.044 (1.031; 1.058) |
| *Coefficients <1 indicates diseconomies of scale, coefficients = 1 indicates constant gains of scale, while coefficients > 1 indicates gains of scale  **Elasticity coefficients | | | |

^*p*-value < 0.001
